# Supplementary material for: Bayesian refinement of protein structures and ensembles against SAXS data using molecular dynamics
Source: PLoS Comput Biol. 2017 Oct 18;13(10):e1005800. doi: 10.1371/journal.pcbi.1005800 (PMC5662244; doi:10.1371/journal.pcbi.1005800)
Supplement: S1 Archive — (BZ2) [file pcbi.1005800.s011.tar.bz2 › gromacs_4.62_bayesian_saxs_refi/share/html/online/g_tune_pme.html]

g\_tune\_pme


| g\_tune\_pme Main Table of Contents |

**VERSION 4.6  
Sat 19 Jan 2013**

---

### Description

For a given number -np or -ntmpi of processors/threads, this program systematically
times mdrun with various numbers of PME-only nodes and determines
which setting is fastest. It will also test whether performance can
be enhanced by shifting load from the reciprocal to the real space
part of the Ewald sum.
Simply pass your .tpr file to g\_tune\_pme together with other options
for mdrun as needed.

Which executables are used can be set in the environment variables
MPIRUN and MDRUN. If these are not present, 'mpirun' and 'mdrun'
will be used as defaults. Note that for certain MPI frameworks you
need to provide a machine- or hostfile. This can also be passed
via the MPIRUN variable, e.g.

export MPIRUN="/usr/local/mpirun -machinefile hosts"

Please call g\_tune\_pme with the normal options you would pass to
mdrun and add -np for the number of processors to perform the
tests on, or -ntmpi for the number of threads. You can also add -r
to repeat each test several times to get better statistics.

g\_tune\_pme can test various real space / reciprocal space workloads
for you. With -ntpr you control how many extra .tpr files will be
written with enlarged cutoffs and smaller Fourier grids respectively.
Typically, the first test (number 0) will be with the settings from the input
.tpr file; the last test (number ntpr) will have the Coulomb cutoff
specified by -rmax with a somwhat smaller PME grid at the same time.
In this last test, the Fourier spacing is multiplied with rmax/rcoulomb.
The remaining .tpr files will have equally-spaced Coulomb radii (and Fourier spacings) between these extremes. **Note** that you can set -ntpr to 1
if you just seek the optimal number of PME-only nodes; in that case
your input .tpr file will remain unchanged.

For the benchmark runs, the default of 1000 time steps should suffice for most
MD systems. The dynamic load balancing needs about 100 time steps
to adapt to local load imbalances, therefore the time step counters
are by default reset after 100 steps. For large systems (>1M atoms), as well as
for a higher accuarcy of the measurements, you should set -resetstep to a higher value.
From the 'DD' load imbalance entries in the md.log output file you
can tell after how many steps the load is sufficiently balanced. Example call:

g\_tune\_pme -np 64 -s protein.tpr -launch

After calling mdrun several times, detailed performance information
is available in the output file perf.out.
**Note** that during the benchmarks, a couple of temporary files are written
(options -b\*), these will be automatically deleted after each test.

If you want the simulation to be started automatically with the
optimized parameters, use the command line option -launch.

### Files

| option | filename | type | description |
| --- | --- | --- | --- |
| **-p** | perf.out | Output | Generic output file |
| **-err** | bencherr.log | Output | Log file |
| **-so** | tuned.tpr | Output | Run input file: tpr tpb tpa |
| **-s** | topol.tpr | Input | Run input file: tpr tpb tpa |
| **-o** | traj.trr | Output | Full precision trajectory: trr trj cpt |
| **-x** | traj.xtc | Output, Opt. | Compressed trajectory (portable xdr format) |
| **-cpi** | state.cpt | Input, Opt. | Checkpoint file |
| **-cpo** | state.cpt | Output, Opt. | Checkpoint file |
| **-c** | confout.gro | Output | Structure file: gro g96 pdb etc. |
| **-e** | ener.edr | Output | Energy file |
| **-g** | md.log | Output | Log file |
| **-dhdl** | dhdl.xvg | Output, Opt. | xvgr/xmgr file |
| **-field** | field.xvg | Output, Opt. | xvgr/xmgr file |
| **-table** | table.xvg | Input, Opt. | xvgr/xmgr file |
| **-tabletf** | tabletf.xvg | Input, Opt. | xvgr/xmgr file |
| **-tablep** | tablep.xvg | Input, Opt. | xvgr/xmgr file |
| **-tableb** | table.xvg | Input, Opt. | xvgr/xmgr file |
| **-rerun** | rerun.xtc | Input, Opt. | Trajectory: xtc trr trj gro g96 pdb cpt |
| **-tpi** | tpi.xvg | Output, Opt. | xvgr/xmgr file |
| **-tpid** | tpidist.xvg | Output, Opt. | xvgr/xmgr file |
| **-ei** | sam.edi | Input, Opt. | ED sampling input |
| **-eo** | edsam.xvg | Output, Opt. | xvgr/xmgr file |
| **-j** | wham.gct | Input, Opt. | General coupling stuff |
| **-jo** | bam.gct | Output, Opt. | General coupling stuff |
| **-ffout** | gct.xvg | Output, Opt. | xvgr/xmgr file |
| **-devout** | deviatie.xvg | Output, Opt. | xvgr/xmgr file |
| **-runav** | runaver.xvg | Output, Opt. | xvgr/xmgr file |
| **-px** | pullx.xvg | Output, Opt. | xvgr/xmgr file |
| **-pf** | pullf.xvg | Output, Opt. | xvgr/xmgr file |
| **-ro** | rotation.xvg | Output, Opt. | xvgr/xmgr file |
| **-ra** | rotangles.log | Output, Opt. | Log file |
| **-rs** | rotslabs.log | Output, Opt. | Log file |
| **-rt** | rottorque.log | Output, Opt. | Log file |
| **-mtx** | nm.mtx | Output, Opt. | Hessian matrix |
| **-dn** | dipole.ndx | Output, Opt. | Index file |
| **-bo** | bench.trr | Output | Full precision trajectory: trr trj cpt |
| **-bx** | bench.xtc | Output | Compressed trajectory (portable xdr format) |
| **-bcpo** | bench.cpt | Output | Checkpoint file |
| **-bc** | bench.gro | Output | Structure file: gro g96 pdb etc. |
| **-be** | bench.edr | Output | Energy file |
| **-bg** | bench.log | Output | Log file |
| **-beo** | benchedo.xvg | Output, Opt. | xvgr/xmgr file |
| **-bdhdl** | benchdhdl.xvg | Output, Opt. | xvgr/xmgr file |
| **-bfield** | benchfld.xvg | Output, Opt. | xvgr/xmgr file |
| **-btpi** | benchtpi.xvg | Output, Opt. | xvgr/xmgr file |
| **-btpid** | benchtpid.xvg | Output, Opt. | xvgr/xmgr file |
| **-bjo** | bench.gct | Output, Opt. | General coupling stuff |
| **-bffout** | benchgct.xvg | Output, Opt. | xvgr/xmgr file |
| **-bdevout** | benchdev.xvg | Output, Opt. | xvgr/xmgr file |
| **-brunav** | benchrnav.xvg | Output, Opt. | xvgr/xmgr file |
| **-bpx** | benchpx.xvg | Output, Opt. | xvgr/xmgr file |
| **-bpf** | benchpf.xvg | Output, Opt. | xvgr/xmgr file |
| **-bro** | benchrot.xvg | Output, Opt. | xvgr/xmgr file |
| **-bra** | benchrota.log | Output, Opt. | Log file |
| **-brs** | benchrots.log | Output, Opt. | Log file |
| **-brt** | benchrott.log | Output, Opt. | Log file |
| **-bmtx** | benchn.mtx | Output, Opt. | Hessian matrix |
| **-bdn** | bench.ndx | Output, Opt. | Index file |

### Other options

| option | type | default | description |
| --- | --- | --- | --- |
| **-[no]h** | bool | no | Print help info and quit || **-[no]version** | bool | no | Print version info and quit || **-nice** | int | 0 | Set the nicelevel || **-xvg** | enum | xmgrace | xvg plot formatting: xmgrace, xmgr or none || **-np** | int | 1 | Number of nodes to run the tests on (must be > 2 for separate PME nodes) || **-npstring** | enum | -np | Specify the number of processors to $MPIRUN using this string: -np, -n or none || **-ntmpi** | int | 1 | Number of MPI-threads to run the tests on (turns MPI & mpirun off) || **-r** | int | 2 | Repeat each test this often || **-max** | real | 0.5 | Max fraction of PME nodes to test with || **-min** | real | 0.25 | Min fraction of PME nodes to test with || **-npme** | enum | auto | Within -min and -max, benchmark all possible values for -npme, or just a reasonable subset. Auto neglects -min and -max and chooses reasonable values around a guess for npme derived from the .tpr: auto, all or subset || **-fix** | int | -2 | If >= -1, do not vary the number of PME-only nodes, instead use this fixed value and only vary rcoulomb and the PME grid spacing. || **-rmax** | real | 0 | If >0, maximal rcoulomb for -ntpr>1 (rcoulomb upscaling results in fourier grid downscaling) || **-rmin** | real | 0 | If >0, minimal rcoulomb for -ntpr>1 || **-[no]scalevdw** | bool | yes | Scale rvdw along with rcoulomb || **-ntpr** | int | 0 | Number of .tpr files to benchmark. Create this many files with different rcoulomb scaling factors depending on -rmin and -rmax. If < 1, automatically choose the number of .tpr files to test || **-steps** | step | 1000 | Take timings for this many steps in the benchmark runs || **-resetstep** | int | 100 | Let dlb equilibrate this many steps before timings are taken (reset cycle counters after this many steps) || **-simsteps** | step | -1 | If non-negative, perform this many steps in the real run (overwrites nsteps from .tpr, add .cpt steps) || **-[no]launch** | bool | no | Launch the real simulation after optimization || **-[no]bench** | bool | yes | Run the benchmarks or just create the input .tpr files? || **-[no]append** | bool | yes | Append to previous output files when continuing from checkpoint instead of adding the simulation part number to all file names (for launch only) || **-[no]cpnum** | bool | no | Keep and number checkpoint files (launch only) |

---

http://www.gromacs.org  
gromacs@gromacs.org
